# Impact of proactive community case management of malaria on malaria prevalence in Bankass, Mali: a cluster-randomised controlled trial

**DOI:** 10.1186/s12936-026-05849-5

**Published:** 2026-05-20

**Authors:** Julie Thwing, Saibou Doumbia, Aliou Traore, Mahamadou Sylla, Mahamadou Sogoba, Idrissa Kamara, Yacouba Samaké, Lamine Guindo, Stephanie Rapp, Emily Treleaven, Calvin Chiu, Caroline Whidden, Aminatou Kone, Abdoulaye A. Djimde, Ari D. Johnson, Kassoum Kayentao

**Affiliations:** 1 Malaria Branch, Division of Parasitic Diseases and Malaria, Centers for Disease Control and Prevention, Atlanta, GA USA; 2 Muso, Bamako, Mali; 3 Malaria Research & Training Centre, Université des Sciences, des Techniques et des Technologies de Bamako, Bamako, Mali; 4 Norwegian Refugee Council, Bamako, Mali; 5 Croix Rouge Malienne, Bamako, Mali; 6 Institute for Social Research, University of Michigan, Ann Arbor, MI USA; 7 Institute for Health and Aging, University of California, San Francisco, San Francisco, CA USA; 8 Department of Disease Control, London School of Hygiene and Tropical Medicine, London, UK; 9 Department of Medicine, University of California, San Francisco, San Francisco, USA

**Keywords:** Community health worker, Malaria, Household visit, Community case management, Proactive

## Abstract

**Background:**

Proactive community case management (ProCCM) of malaria is a strategy to improve access to prompt and effective case management of malaria at the community level by supporting community health workers (CHWs) to visit every household in their community every 1 to 2 weeks to identify people with malaria symptoms, offer rapid diagnostic tests, and first line malaria treatment for those with positive tests. We sought to determine if this strategy could decrease malaria parasite prevalence in high malaria transmission settings.

**Methods:**

CHWs in the intervention (ProCCM) arm were asked to visit households proactively twice per month to offer an extensive package of maternal and child health services, including malaria case management for males and females of all ages, compared to those in the control arm that offered these services from a fixed point in the community. We measured parasite prevalence among all ages, and fever prevalence, care seeking, and access to diagnostic testing among children under 5 years at the endline survey of a 3-year cluster-randomized controlled trial of ProCCM, covering a population of over 100,000 in Bankass, Mali, a remote rural area that became a conflict zone during the study period.

**Results:**

There was no difference between intervention and control arms in parasite prevalence among all ages, fever in the last 2 weeks, care seeking, or access to diagnostic testing for malaria among children under 5 years. However, CHWs in the intervention arm reached the goal of two visits per month in less than half the households.

**Conclusions:**

In this high transmission setting, with well-supported, supplied, supervised, and compensated CHWs, both fixed point (control) and proactive household (intervention) visits offered comparable benefits in extending access to malaria case management to community members, despite intervention arm CHWs not reaching the target household visit frequency.

*Trial registration* Trial registration number NCT02694055 (registered February 26, 2016).

## Background

Globally, the all-cause under-five mortality (U5M) rate has declined by 60% from 1990 to 2022, from 93 to 37 deaths per 1000 live births. Sub-Saharan Africa (SSA) accounts for 55% of under-five deaths, with pneumonia (17%), malaria (15%), and diarrhea (11%) accounting for more than 40% of all under-five deaths in the region [1]. Integrated community case management (iCCM) was developed to increase access to care for these conditions, with the aim of decreasing mortality from these killers. However, evidence on iCCM scale-up to date has not consistently supported a decrease in mortality and has not assessed malaria prevalence as an outcome [2].

The World Health Organization (WHO) recommends supporting community health workers (CHWs) through remuneration, supply chain management, supportive supervision, and functional referral systems [3]. However, even with iCCM programs in place, barriers to care remain, and healthcare access may be poor [4, 5]. In most iCCM programs, CHW workflow is passive, relying on community members to seek care [6].

Active fever surveillance, involving regular household visits to detect and treat malaria infections among residents with febrile illness, has been a part of numerous malaria control/elimination programs in Latin America and Asia [7–9]. However, a systematic review of proactive case detection found uncertain evidence for the effectiveness of proactive case detection on the prevalence of infectious diseases in children [10]. Proactive household visits, in which CHWs conduct regular visits to every household in the community to identify prospective patients and offer preventive or curative services, have been studied in several SSA countries. While some have offered iCCM or an expansive package of maternal and child health care [11], others have focused on malaria [12, 13]. In Senegal, in an area of moderate seasonal malaria transmission, a cluster-controlled quasi-experimental trial of proactive community case management (ProCCM) found that communities that received weekly household visits to identify people with febrile illness, offer a rapid diagnostic test (RDT), and treat those with positive results had a 16-fold lower point prevalence of symptomatic, parasitologically confirmed malaria infection at the end of 20 weeks of visits, compared to communities in which a resident CHW offered malaria community case management (CCM) to those who sought care, but was not conducting weekly household visits [12]. When scaled up to four regions, CHWs offering ProCCM detected and treated 300% more malaria cases than they had prior to the program, prompting scale-up to 10 of Senegal’s 14 regions [14]. In Madagascar, a cluster-randomized controlled trial of ProCCM visits every 2 weeks demonstrated decreased malaria parasite prevalence among children 5–14 years compared to routine iCCM (odds ratio 0.59; 95% CI [0.38–0.91]) [13].

A 7-year repeated cross-sectional study in peri-urban Bamako documented an increase in access to effective antimalarial treatment within 24 hours of symptom onset from 14.7% in 2008 to 35.3% in 2015, and a 55% relative reduction in febrile illness during the previous 2 weeks among children under 5 years [11]. However, this study was uncontrolled and included a comprehensive package of services, including reinforcing training and care at health facilities and user fee removal, making it impossible to isolate the effect of proactive home visits.

## Methods

We sought to determine if ProCCM by CHWs reduces malaria prevalence, with a primary outcome of parasite prevalence by RDT among all ages. We integrated measurement of malaria prevalence into a previously described 3-year cluster-randomized controlled trial in Bankass, Mopti, in rural Mali, that compared a comprehensive package of maternal and child health services delivered through CHWs using a proactive workflow of regular household visits compared to the same package of interventions delivered through a passive workflow by CHWs based at fixed community health sites (dependent on care seeking by individuals) [15]. To measure parasite prevalence, we integrated collection of RDTs and filter paper into the endline survey. We also used data from the care-seeking portions of the endline survey, as well as data collected by CHWs during their patient consultations, to measure differences in access to care for malaria.

### Trial design

We conducted a cluster-randomized controlled trial, with a stratified, two-arm, parallel group design, in the Bankass health district of the Mopti region in central Mali (ClinicalTrials.gov NCT02694055), described in detail elsewhere [15, 16]. The Bankass health district is rural, with high parasite prevalence (26.6% among children 6–59 months in Mopti), high under-five mortality and low healthcare utilization at baseline [17–19]. Communities in seven primary health facility catchment zones (covering a population of greater than 100,000) were mapped prior to the baseline survey. The 137 clusters in the trial comprised single villages or villages and hamlets less than one kilometer apart in order to reduce contamination. A map of the study area was published in the study protocol [15]. The randomization scheme was stratified along two dimensions: health catchment area and distance to the nearest PHC (< 1.0 km, 1.0–5.0 km, and > 5.0 km). Using a computer-generated random allocation, village clusters within each strata were randomly assigned to the intervention or control arm by an investigator based in the United States who did not have any contact with study implementation. Clusters were randomized 1:1 to CHW care delivered via proactive home visits (69 intervention clusters) or at a fixed community health site in the cluster (68 control clusters). Clusters had one or more CHWs to maintain CHW:population ratios of approximately 1:700 in accordance with Mali’s national iCCM strategy. Prior to the baseline survey, all households in the study area were enumerated, with all permanent household residents listed in a household roster. At follow-up surveys at 12, 24, and 36 months, all new households and household residents were also eligible to participate in the survey. All permanent residents and visitors in the study area were eligible to receive healthcare services. While it was not possible to blind participants, providers, and survey personnel during the study, trial statisticians remained blinded throughout the trial.

### Intervention implementation

All CHWs in both arms were selected, salaried, trained, supervised, and supplied in line with WHO recommendations, as described elsewhere [15, 16]. In the intervention arm, CHWs were instructed to spend 2 h daily, 6 days per week, conducting proactive household visits, with the goal of at least twice monthly household visits. In the control arm, CHWs were instructed to spend 4 h per day, 6 days per week, at fixed sites in the community. CHWs in both trial arms provided the same package of services, including iCCM, and were available to provide care at any time as needed. Residents of all ages who reported fever or symptoms consistent with malaria during the last 2 days were to receive testing for malaria with a histidine-rich protein II (HRP-2) based RDT, with treatment with artemisinin-based combination therapy (ACT) for those with positive RDT results. All patients, including those with negative RDT results, were also evaluated and treated for other conditions according to the iCCM protocol. Patients with danger signs were referred to the nearest primary health facility, and offered transport if available.

All CHWs received salaries and benefits corresponding to local minimum wage requirements, with performance-based opportunities for advancement. All CHWs received individual monthly supervision and twice monthly group supervision meetings with dedicated community supervisors. Supervisors monitored CHW supplies and equipment, including the CHW smartphone mobile application for recording all patient encounters. All primary healthcare facilities received infrastructure reinforcements, and user fees were removed for patients seeking care. The CHW program launched in February 2017.

### Data collection

Patient assessments performed by CHWs in both arms were captured in a mobile application supported by Medic Mobile from February 2017 through March 2020 [20].

Household surveys took place December 2016–January 2017 (baseline), February–March 2018, March–May 2019, and February–April 2020 (endline). All households in the study area were eligible for inclusion in each survey, and all individuals in participating households were enumerated in a household roster. All women 15–49 years responded to a questionnaire for women of childbearing age. Survey instruments were based on the Demographic and Health Survey (DHS) and included modules on socioeconomic and demographic characteristics, women’s reproductive health, lifetime birth histories, and recent symptoms and service utilization for children under 5 years of age. Household socioeconomic status was measured by deriving wealth quintiles from an index of durable goods, livestock, and physical housing characteristics using principal components analysis.

### Malaria sub-study

The primary outcome for the malaria sub-study was parasite prevalence among the population as a whole, and stratified by risk group: children < 5 years, children 5–14 years, pregnant women, and non-pregnant individuals 15 years and older, measured by integrating collection of RDTs and filter papers for randomly selected participants of all ages in the endline survey. In order to determine whether the intervention had impacted access to malaria case management, we analyzed data from the women’s survey related to fever and care seeking for fever during the previous 2 weeks among all their children under 5 years of age. We also analyzed data from the CHW mobile application related to RDTs performed (for all ages) in each arm throughout the study period.

### Malaria sub-study sampling

We calculated the sample size for each group of interest (children < 5 years, children 5–14 years, non-pregnant individuals ≥ 15 years, pregnant women) to give 80% power, alpha = 0.05, design effect of 2, and k = 0.1 to detect a difference between study arms at endline in percent parasitemia of 15% vs 10%, assuming sampling was evenly distributed among clusters. This resulted in a total of 5250 individuals per group (2625 per group per arm).

Given that fewer women were expected to be pregnant than the target sample size per group, all women reporting pregnancy at the time of the survey were asked to participate (we estimated 1800 to 2500 women were likely to report being currently pregnant); these data are reported elsewhere (manuscript in preparation).

Using unique individual identification numbers from the 24-month census, we selected a simple random sample for each group of interest (children < 5 years, children 5–14 years, non-pregnant individuals ≥ 15 years). Of note, this excluded most children < 12 months at the time of the survey but assured a random sample of those who had been resident in the study area for at least the past year. For those ≥ 15 years, we selected 5500 (2750 per arm) to offset approximately 250 of the selected group likely to report being pregnant.

### Malaria sub-study survey procedures

The endline survey instruments flagged anyone who had been pre-selected for blood testing during the simple random selection (as well as all women reporting current pregnancy). For these, the interviewer explained their selection and the blood testing process and obtained informed consent. Participants were requested to report to the laboratory station at a central location in the community. For anyone who was absent, materials were left requesting participation and directing them to the testing location.

All selected consenting participants presenting to the laboratory station received a finger prick to perform the RDT rapid diagnostic test for malaria. All participants with a positive RDT were treated with ACT in accordance with the Mali Ministry of Health policy unless they were asymptomatic and had received treatment within the last 2 weeks. A small amount (< 1 ml) of blood was collected from children < 5 years of age and pregnant women on Whatman 903 filter paper, then dried and placed in plastic bags with desiccant.

### Laboratory processing

Polymerase chain reaction (PCR) confirmation was performed on dried blood spots (DBS) collected during the endline survey for all children < 5 years with positive RDTs, and 10% of those with negative RDTs. Total deoxy-ribonucleic acid (DNA) was extracted using the dried blood spot extraction protocol of Qiagen Kit® (QIAgen, Hilden, Germany). We performed a nested PCR amplification using two pairs of primers targeting the *pfdhfr* gene. A first amplification was done with 5 µl of the extracted DNA with a first pair of primers:

Sense primer FR51-A (GCGCGCTAATAACTACACATTTA), Antisense primer FR51-B (CCCGGGCTCTTATATTTCAATTT).

A PCR product of 147 bp was obtained and used to do a secondary amplification using the following primers: Sense primer FR51-D (CTAGGAAATAAAGGAGTATTACCATGGAAATGGA), Antisense primer FR59-D (ATTTTTCATATTTTGATTCATTCACATATGTTGTAACTGTAC). A final PCR product of 113 bp was obtained and migrated on agarose gel. All samples presenting an amplicon at 113 bp were considered PCR-positive for *Plasmodium falciparum*.

### Ethics

Any individual, regardless of study enrollment status, permanent residency, study arm, or age, who sought health care from study CHWs were eligible to receive care. The ProCCM trial received ethical approval from the ethics committee at the Université des Sciences, des Techniques et des Technologies of Bamako (USTTB) (Ref: 2016/03/CE/FMPOS). The malaria sub-study was added as a protocol amendment and approved by the ethics committee at USTTB. CDC was non-engaged. All participants provided written informed consent for the household survey. Adults 18 years and older and married women less than 18 years of age completed a separate written informed consent for their own participation in the RDT for malaria, as well as for their children under 18 years of age. Children and adolescents ages 12 to 17 provided written assent for their participation in the malaria RDT.

### Data management

Data were analyzed using SAS 9.4 (Cary, NC). Frequencies, odds ratio estimates, and confidence intervals were adjusted for clustering using robust variance estimation. Logistic regression models adjusted for parasite prevalence, socioeconomic status (wealth quintile), cluster population size at study baseline, and distance from the nearest primary health facility (Euclidean distance in kilometers estimated via GPS). Due to the simple random sampling scheme, the analysis was unweighted. These analyses were conducted using an intention-to-treat (ITT) approach. We also estimated the per protocol (PP) effect of the intervention at the household level as follows: using the dataset from the CHW mobile application, for the intervention arm, treatment adherence was defined as receiving two or more home visits from a CHW in the month preceding the survey. In the control arm, adherence was defined as receiving no home visits in the month preceding the survey. Parasite prevalence by RDT in the endline survey among the general population was estimated for individuals living in households defined as adherent in each arm.

Using the dataset from the CHW mobile application, the number of RDTs performed and the number of positive RDTs recorded by CHWs per month were calculated over the 3 year period of the study. The number of RDTs performed and the RDT positivity rate in each arm were aggregated by cluster and calculated for each month of the study and overall. We plotted the number of RDTs per month and RDT positivity rate by arm and examined whether there was a difference in means for each indicator by treatment arm using t-tests.

#### Patient and public involvement

The ProCCM Trial was designed and implemented in partnership with national, district and local health officials of the Malian Ministry of Health with patient and public engagement described in detail elsewhere [21]. For the malaria sub-study reported here, we engaged with communities when we conducted outreach in parallel with the annual engagement activities prior to the launch of that year’s household survey. We described the specific malaria sub-study procedures, and sought community consultation and permission prior to the launch of the sub-study in meetings with administrative representatives of study villages. We also consulted with community members including religious leaders and women’s and youth groups. These groups then communicated with other community members via open public meetings. Trial findings have been disseminated via workshops at all levels of local, regional, and national representation.

## Results

Of the 15,750 pre-selected individuals, 12,554 participated in the malaria sub-study (4580 children < 5 years—participation 87%, 4585 children 5–14 years—participation 87%, and 3389 non-pregnant individuals ≥ 15 years—participation 65%); non-participation was primarily due to individuals being pre-selected but not present at the time of interview. The 1848 women reporting current pregnancy participated and are reported elsewhere. Most non-participating pre-selected individuals were absent. Males made up 51.0% (95% CI 49.6–52.5), 50.9% (95% CI 49.4–52.3), and 42.4% (95% CI 40.8–44.1) of children < 5 years, children 5–14 years, and non-pregnant individuals, respectively. There was no significant difference between study arms in sociodemographic characteristics (Table 1).

**Table 1:** Characteristics of malaria sub-study participants by arm

	Intervention	Control
Number of participants in malaria sub-study	7418	6984
< 5 years	2316 (31.2%)	2264 (32.4%)
5–14 years	2380 (32.1%)	2206 (31.6%)
≥ 15 years	2722 (36.7%)	2514 (36.0%)
Percent male	42.3%	42.5%
Socioeconomic quintile		
1 (Least poor)	1009 (13.6%)	942 (13.5%)
2	1344 (18.1%)	1263 (18.1%)
3	1504 (20.3%)	1465 (21.0%)
4	1737 (23.4%)	1642 (23.5%)
5 (Poorest)	1824 (24.6%)	1672 (23.9%)
At least 5 km from health facility	4172 (56.2%)	3526 (50.5%)
Community population > 700	5221 (70.4%)	4418 (63.3%)
Mothers/guardians with no formal education	1378/1581 (87.2%)	1258/1488 (84.5%)

There was no significant difference in parasite prevalence by RDT between study arms for any age group in univariate analysis (Table 2). The multivariate analysis showed no difference when adjusted for maternal education, socioeconomic status (wealth quintile), distance from facility (< 5 km vs > 5 km), and cluster population above or below 700.

**Table 2:** Parasite prevalence by study arm and age group, Bankass, Mali, 2020

Age group	Intervention % (95%CI)	Control % (95%CI)	OR (95% CI)
< 5 years	26.0% (22.3–29.7)	25.4% (21.2–29.6)	1.03 (0.77–1.38)
5–14 years	49.0% (45.1–52.8)	53.0% (49.2–56.9)	0.85 (0.69–1.06)
≥ 15 years (non-pregnant)	20.6% (18.4–22.7)	17.9% (15.0–20.8)	1.19 (0.94–1.50)
All	30.7% (28.1–33.4)	31.4% (28.3–34.5)	0.97 (0.80–1.17)

PCR results for children < 5 years showed good concordance with RDT results, particularly for negative RDTs. Among the 1259 children < 5 years with positive RDTs for whom PCR results were available, 82.4% (1037) were PCR-positive, consistent with recently cleared infections among some children. Among the 10% of RDT-negative children for whom PCR confirmation was done, 95.6% (345/361) were PCR-negative, indicating remarkably few malaria infections detectable by PCR that were not detectable by RDT among children under 5 years.

Endline survey data on fever and care seeking in the previous 2 weeks were available for 4290 (93%) malaria sub-study children < 5 years. Among these children, 16.4% (95% CI 15.0–17.8) in the intervention arm and 15.4% (95% CI 13.6–17.2) in the control arm reported fever in the previous 2 weeks. Of these, 63.8% (n = 351, 95% CI 58.8–68.8) in the intervention arm and 65.9% (n = 323, 95% CI 60.0–71.9) in the control arm had sought care with a qualified provider (including a CHW). Of children who had sought care, 79.2% (95% CI 73.0–85.4) in the intervention arm and 82.7% (95% CI 76.1–89.3) in the control arm had sought care with a CHW. Overall, among those reporting fever in the previous 2 weeks, 49.8% (95% CI 44.4–55.0) in the intervention arm and 50.9% (95% CI 44.3–57.6) in the control arm had received a diagnostic test for malaria. In both arms combined, 76.7% (95% CI 72.0–81.4) of those who sought care with a CHW received a diagnostic test, compared to 62.1% (95% CI 50.8–73.3) who had sought care, but not from a CHW.

**Fig. 1:**
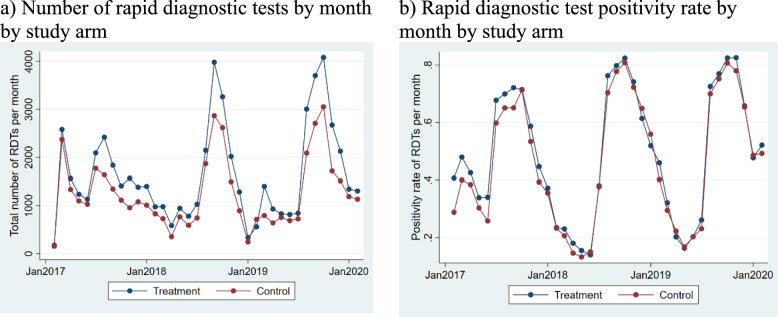
**a** Number of rapid diagnostic tests by month by study arm. **b** Rapid diagnostic test positivity rate by month by study arm

Throughout the trial period, (February 2017–March 2020), the CHW application captured 219,410 patient assessments, containing data from 80,913 individuals in 19,773 households, during which 107,428 RDTs were conducted, 57% of which were performed in intervention arm clusters (mean 26.5 RDTs per cluster monthly in the intervention arm and 21.6 RDTs per cluster monthly in the control arm). The difference in the number of RDTs between the study arms occurred primarily during the malaria transmission season (August-November) (Fig. 1a), but the RDT positivity rate (62,271 positive RDTs, overall positivity rate 57.9%) did not vary by study arm (Fig. 1b).

The per-protocol analysis demonstrated that 39% of individuals in the intervention arm lived in households that had received ≥ 2 visits in the month before the endline survey, while 79% in the control arm had not received a household visit in the year prior to the survey (defined as adherent to treatment arm). The per protocol analysis showed no difference in parasite prevalence. Among households that met the per protocol definition, RDT positivity was 32.7% (95% CI 28.8–36.6) in the control arm vs 29.6 (95% CI 26.7–32.6) in the intervention arm.

## Discussion

There was no difference in malaria prevalence at 36 months between communities in which the CHWs conducted regular door to door visits including malaria case management for symptomatic residents, compared to the same services offered by CHWs at fixed village sites. Additionally, there was no difference between arms at 36 months in the proportion of children with fever in the previous 2 weeks, the proportion of those febrile children for whom care was sought, and the proportion who received a diagnostic test for malaria, as reported by their guardians. Program data from the CHW mobile application, while showing that proactive CHWs conducted somewhat more RDTs than fixed-site CHWs during the transmission season, overall showed a high degree of similarity between the arms in terms of care delivery by CHWs.

The combination of supportive interventions that was implemented in both arms—including training on a comprehensive package of maternal and child-care services, dedicated supervision, compensation, and supply chain support for CHWs, as well as reinforcement of primary healthcare teams and infrastructure and user fee removal—dramatically increased access to care [22] and decreased all-cause under-five mortality [16] at the end of the trial period compared to baseline. However, data from the mobile application suggested that less than half of households in the intervention arm received the prescribed two household visits per month, so the trial was not able to assess the impact of the intervention as intended. The reasons why intervention CHWs failed to meet these targets could have varied across clusters, such as intervention fatigue, opportunity costs relative to offering fixed-site care, a perceived reduced value once a CHW established relationships and protocols with the community, or perceived threats related to armed conflict in the study area. While it was not directly measured, our process evaluation respondents described good adherence to the protocol [23], suggesting that lower population:CHW ratios are needed to meet home visiting targets. The malaria-specific results are consistent with the overall results of the trial on the primary and secondary outcomes of under-five mortality and utilization of child health services. The trial found no difference in all-cause child mortality between arms, though there was a greater than 60% pre-post reduction, from 148.4 to 60.1 deaths per 1000 live births from baseline to endline [16]. Overall utilization of child health services for fever, cough, or diarrhea among children under 5 years increased from 19% at baseline to 61% at 12 months across all clusters, before declining to 44% at 24 months, and then rising to 52% at 36 months. While access to care was higher in the intervention arm at 12 months, there was no difference at 24 and 36 months [22]. Of note, the study area was impacted by violent conflict between the 12 and 36 month time points, displacing many residents and greatly increasing the challenge in delivering care in both arms [24]. Despite the dramatic increase in access to care and decrease in all-cause under-five mortality in both arms over the course of the study, the overall 26% parasite prevalence by RDT among children under 5 years in the study area is very similar to the 26.6% parasite prevalence reported in Mopti region in the 2021 Malaria Indicator Survey [19].

These results contrast with those reported from Senegal, where weekly proactive household visits for malaria case management reduced point prevalence of fever and symptomatic malaria infection compared to routine malaria CCM for all ages [12], and Madagascar, where parasite prevalence decreased more in the proactive arm (household visits every 2 weeks) compared to routine malaria CCM for children under 5 years [13]. However, in Senegal, routine malaria CCM (in both arms) and weekly proactive household visits (intervention arm) were available for all ages, and in Madagascar, while the proactive household visits offered malaria case management for all ages every 2 weeks in the intervention arm, routine malaria CCM (in both arms) was only offered to children under 5 years. In Mali, both arms offered routine CCM to all age groups, though the proactive household visits, intended to be every 2 weeks, did not reach this goal in the majority of intervention-arm households. There was a relatively high degree of fidelity to intervention implementation reported in Senegal (89% of sweeps completed) and Madagascar (80–100% of households visited on average weekly, and 60–95% of households agreed to the visit each week). However, it is not possible to directly compare these metrics. Both Senegal and Madagascar reported several-fold increases in the number of RDTs performed and cases diagnosed and treated at the community level in the intervention arm, whereas the number of RDTs performed in Mali was relatively similar between arms. While CHWs in both arms received training, supply chain support, and supervision in Senegal, Madagascar, and Mali, both arms in Mali also received substantial health systems strengthening support (removed user fees, support for infrastructure). In addition, CHWs in the control arm in Mali were instructed to spend a fixed number of hours per day at a central point in the community, whereas this was not a requirement in the control arm in Senegal or Madagascar. Finally, while transmission is highly seasonal in Senegal, Madagascar, and Mali, it is substantially higher in Mali, which thus theoretically has a higher proportion of asymptomatic infections than in Senegal and Madagascar. Artemisinins have gametocytocidal properties; gametocytes persist on average 55 days after treatment with a non-ACT but 13 days after treatment with an ACT [25]. Modelling predicts that treatment of symptomatic malaria infection with ACT compared to non-ACT would result in decreased parasite prevalence [26], and that very high coverage (93–98%) of prompt (≤ 5 days) ACT treatment of symptomatic malaria infections in low transmission settings could interrupt transmission [27]. Offering case management to symptomatic individuals, however, neglects those with asymptomatic parasitemia, and the likelihood of fever decreases with increasing age and transmission levels [28]. The proportion of malaria infections that are asymptomatic decreases as parasite prevalence decreases, but even at low transmission, upwards of 60% of infections may be asymptomatic [29].

This study faced substantial limitations. The effect of having reached the target frequency of household visits (twice monthly) in less than half of households in the intervention arm, and having achieved high and approximately equal coverage of prompt case management in both arms, effectively delivered the intervention equally in both arms, biasing toward the null. Parasite prevalence was integrated only into the endline survey, so it is impossible to rule out a baseline difference between arms (though the randomization process makes this unlikely), or to demonstrate a pre-post difference. A substantial proportion of those selected to participate, particularly among those 15 years and older (who were absent and may have been working elsewhere or displaced due to conflict), did not report to the laboratory station and thus had missing data. However, there was no evidence of participation bias by arm. The process evaluation data collection did not reveal why meeting the target for visit frequency was not reached.

These findings highlight the need for further investigation in several areas. First, while comprehensive reinforcement of CHWs and facility-based primary care resulted in major increases in access to care and decreases in child mortality, because the intervention of twice monthly household visits was not achieved, it was not possible to measure the impact of the intervention as intended on parasite prevalence. While this study was intended to measure the impact of proactive household visits compared to fixed hours at a central location in the community, most CHW programs do not in practice have fixed hours and CHWs may be difficult to locate, making it important to measure the impact of proactive visits against the “status quo.” It may be that regular CHW availability is the key ingredient, whether delivered by proactive visits or fixed hours and location. Programmatic feasibility of weekly or twice monthly visits may be influenced by population covered, size of households, distance between households, and ability to find people at home, whether influenced by communal rhythms or conflict. The mix of these elements that creates an enabling environment for implementation, and thus feasibility in a given scenario, remains unknown. The outcome of parasite prevalence may not be appropriate in all settings. In lower transmission settings, it may be decreased more rapidly by relatively good coverage of prompt effective case management, while in higher transmission settings, coverage may need to be impossibly high for a longer period, making severe disease and other clinical outcomes more useful. Finally, future trials should monitor and measure processes and implementation fidelity closely in intervention and control arms, possibly incorporating time-motion study methodologies to understand and optimize delivery; ensure that outcomes are selected to match local epidemiology; and while it is not possible to control for outbreaks of conflict, rigorously assess contextual factors affecting implementation effectiveness.

## Conclusions

In the context of user fee removal, professional CHWs, and upgraded primary care, prompt recommended case management of malaria was comparably high in both arms of this trial, though the goal of two visits per household per month in the intervention arm was not reached. Under these conditions, the addition of proactive home visits did not reduce malaria parasite prevalence. Further research could explore the relationship between parasite prevalence, home visit intensity, and implementation context. In the context of user fee removal, professional CHWs, and upgraded primary care, both trial arms achieved comparable access to malaria case management, with no difference in parasite prevalence at endline. However, critical limitations affect interpretation of these findings.

## Data Availability

Data are available upon reasonable request to the study PI. Please contact Ari Johnson at ajohnson@musohealth.org.

## Abbreviations

ProCCM: Proactive community case management
CHWs: Community health workers
U5M: All-cause under-five mortality
SSA: Sub-Saharan Africa
iCCM: Integrated community case management
WHO: World Health Organization
RDT: Rapid diagnostic test
CCM: Community case management
HRP-2: Histidine-rich protein II
ACT: Artemisinin-based combination therapy
DHS: Demographic and Health Survey
PCR: Polymerase chain reaction
DNA: Deoxy-ribonucleic acid
USTTB: Université des Sciences, des Techniques et des Technologies of Bamako
ITT: Intention-to-treat
PP: Per protocol
